# Simultaneous Renal and Splenic Infarctions Associated with Newly Diagnosed Atrial Fibrillation

**DOI:** 10.7759/cureus.112067

**Published:** 2026-07-04

**Authors:** Kursat K Kerimoglu, Firat Dolas, Burcu T Demirdoven

**Affiliations:** 1 Department of Emergency Medicine, Alsancak Nevvar-Salih Isgoren State Hospital, Izmir, TUR

**Keywords:** atrial fibrillation, emergency department, renal infarction, splenic infarction, systemic embolization

## Abstract

Renal and splenic infarctions are uncommon causes of acute abdominal, flank, or back pain and may be overlooked because of their non-specific presentation. Atrial fibrillation is an important cause of systemic embolization; however, simultaneous renal and splenic infarctions associated with newly diagnosed atrial fibrillation are rarely reported.

A 77-year-old woman presented to the emergency department with acute-onset epigastric, back, and flank pain. She had hypertension and diabetes mellitus. Physical examination revealed diffuse abdominal tenderness without guarding or rebound. Electrocardiography showed newly diagnosed atrial fibrillation. Laboratory tests revealed mild leukocytosis, markedly elevated lactate dehydrogenase (LDH), mildly elevated C-reactive protein, and slightly elevated D-dimer, while serum creatinine was normal. Contrast-enhanced abdominal computed tomography demonstrated a 5-cm non-enhancing splenic lesion and a 2-cm non-enhancing hypodense lesion in the right kidney, consistent with splenic and renal infarctions. CT angiography showed no significant arterial stenosis, mural thrombus, or aortic plaque formation. Anticoagulation with subcutaneous enoxaparin was initiated.

Newly diagnosed atrial fibrillation may be associated with visceral embolic events. In patients with unexplained acute abdominal, back, or flank pain, the coexistence of elevated LDH and atrial fibrillation should prompt consideration of renal and splenic infarctions and early contrast-enhanced imaging.

## Introduction

Acute renal infarction and splenic infarction are uncommon causes of acute abdominal, back, or flank pain [1]. Their diagnosis is frequently delayed or missed because the clinical presentation is non-specific and may mimic more common conditions such as renal colic, acute pyelonephritis, appendicitis, pancreatitis, or other causes of acute abdomen [2,3]. Laboratory abnormalities, including elevated lactate dehydrogenase, leukocytosis, hematuria, or increased inflammatory markers, may provide diagnostic clues; however, these findings are generally non-specific [4,5]. Therefore, contrast-enhanced CT remains central to confirming the diagnosis [5,6].

Cardioembolic events are among the principal causes of visceral infarction [7]. Although atrial fibrillation is traditionally associated with ischemic stroke, systemic embolization may also involve the renal, splenic, mesenteric, or peripheral arterial circulations [8]. Concurrent renal and splenic infarctions are uncommon and should prompt consideration of an underlying systemic embolic or hypercoagulable disorder [2].

Herein, we report a case of simultaneous renal and splenic infarctions diagnosed in the emergency department in a patient with newly diagnosed atrial fibrillation. This case is presented to emphasize that visceral infarctions may occur with non-specific abdominal, back, or flank pain and may therefore be overlooked during the initial emergency evaluation. 

## Case presentation

A 77-year-old woman presented to the emergency department with acute-onset epigastric, back, and flank pain that began earlier the same day. Her medical history included hypertension and diabetes mellitus. According to her medical records, she had no prior diagnosis of atrial fibrillation.

**Figure 1:**
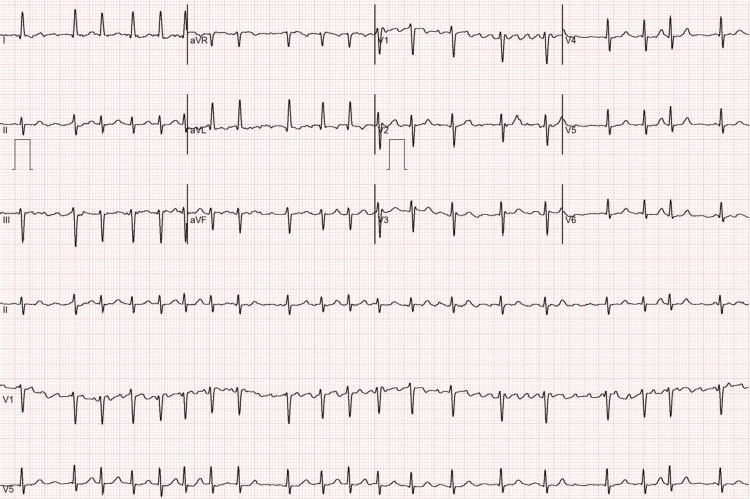
Electrocardiography of the patient on presentation to the emergency department. Twelve-lead electrocardiography obtained on presentation to the emergency department demonstrating atrial fibrillation with a ventricular rate of approximately 110 beats per minute. Organized P waves are not clearly discernible, and variable R–R intervals are observed.

Upon admission, her blood pressure was 110/70 mmHg, heart rate was 110 beats per minute, oxygen saturation was 99% on room air, and body temperature was 36.3°C. She was conscious, cooperative, and oriented, presenting a Glasgow Coma Scale score of 15. No complaint of palpitations was documented at presentation. Physical examination revealed diffuse abdominal tenderness without guarding or rebound tenderness. Peripheral pulses were palpable and symmetric. Electrocardiography demonstrated atrial fibrillation with a rapid ventricular response, which was recorded as newly diagnosed during the emergency department evaluation (Figure 1).

Laboratory findings at presentation are summarized in Table 1. The main abnormalities were mild leukocytosis and neutrophilia, markedly elevated lactate dehydrogenase (LDH), mildly elevated C-reactive protein, slightly elevated D-dimer, and mild elevations in aminotransferase levels.

**Table 1:** Laboratory findings at presentation

Parameter	Result	Reference range
White blood cell count	11.29 × 10³/µL	4.0–10.0 × 10³/µL
Neutrophil count	9.01 × 10³/µL	2.0–7.0 × 10³/µL
Hemoglobin	16.6 g/dL	12.0–16.0 g/dL
Platelet count	219 × 10³/µL	150–450 × 10³/µL
Lactate dehydrogenase	742.2 U/L	109–245 U/L
C-reactive protein	14.3 mg/L	0–6 mg/L
D-dimer	0.67 mg/L FEU	0–0.5 mg/L FEU
Creatinine	0.90 mg/dL	0.7–1.2 mg/dL
Total bilirubin	0.83 mg/dL	0.2–1.1 mg/dL
Direct bilirubin	0.15 mg/dL	0.01–0.25 mg/dL
Aspartate aminotransferase	48.8 U/L	8–40 U/L
Alanine aminotransferase	45.9 U/L	5–40 U/L
Amylase	60 U/L	0–220 U/L

Urinalysis showed high specific gravity, trace blood, 1+ leukocyte esterase, 3+ glucose, and 2+ ketones. Microscopic examination revealed six leukocytes and two erythrocytes, while nitrite and bacteriuria were absent. 

**Figure 2:**
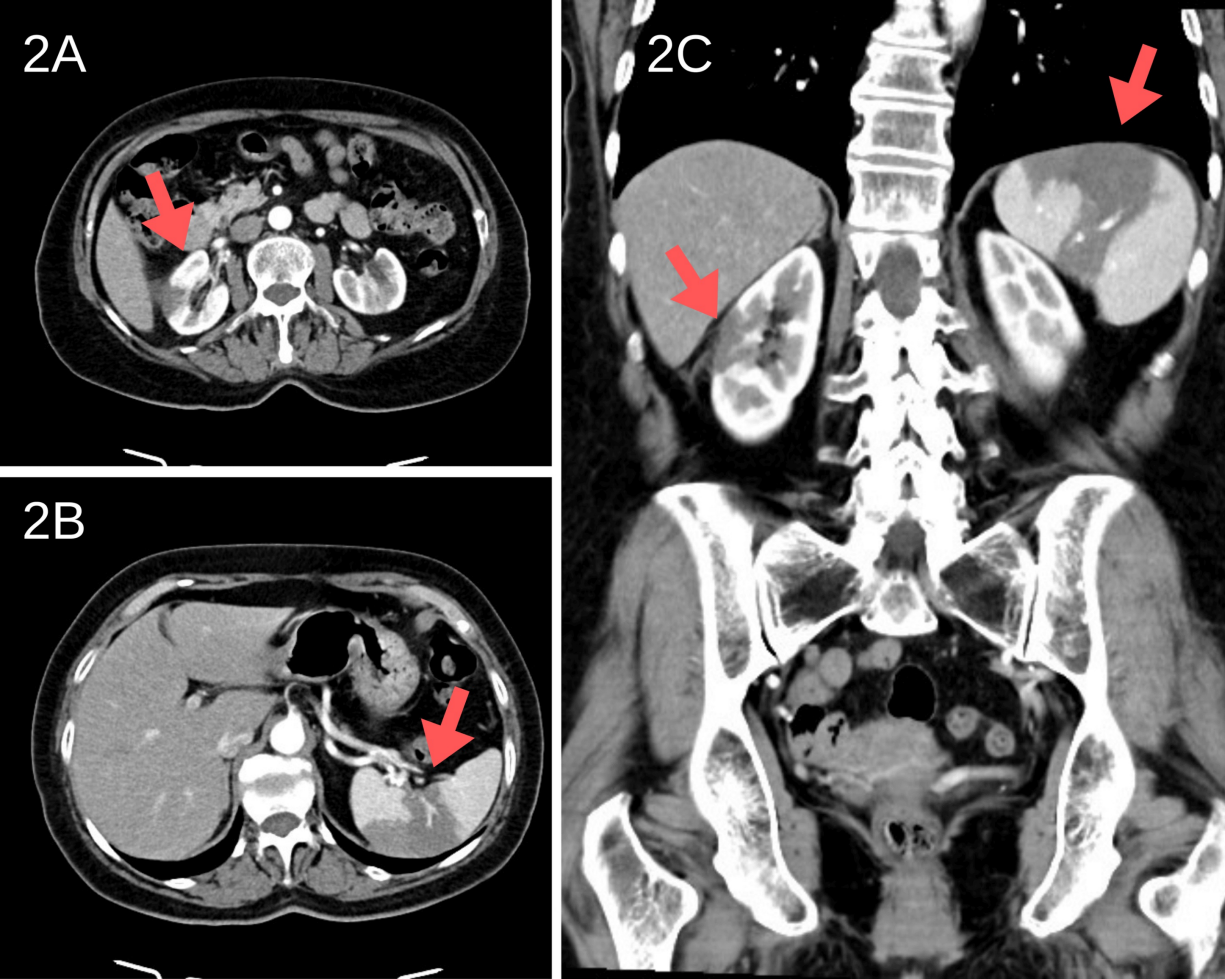
Contrast-enhanced abdominal CT images demonstrating renal and splenic infarctions. (2A) Axial image showing a wedge-shaped non-enhancing hypodense area in the lateral mid-portion of the right kidney, representing a perfusion defect consistent with renal infarction (arrow). (2B) Axial image showing a non-enhancing hypodense area in the spleen, consistent with splenic infarction (arrow). (2C) Coronal reconstruction demonstrating simultaneous right renal and splenic perfusion defects. Red arrows indicate the infarcted areas.

Because the patient had persistent abdominal, back, and flank pain without a clear alternative diagnosis after initial clinical, laboratory, and urinary evaluation, a contrast-enhanced abdominal CT was obtained during the emergency department assessment. Contrast-enhanced abdominal CT demonstrated a 5-cm patchy non-enhancing area in the central portion of the spleen, suggestive of splenic infarction. In addition, a 2-cm non-enhancing hypodense lesion was detected in the lateral mid-zone of the right kidney, suggestive of renal infarction (Figure 2). No hydronephrosis, urinary stone, intra-abdominal free fluid, or free air was detected. 

To further evaluate the arterial system, abdominal CT angiography was performed. No significant stenosis was detected in the splenic artery or right renal artery. No significant plaque formation or mural thrombus was identified in the abdominal aorta. The abdominal aorta, bilateral common iliac arteries, internal and external iliac arteries, celiac trunk, superior mesenteric artery, and bilateral renal arteries were reported to be of normal caliber.

Transthoracic echocardiography revealed a left ventricular ejection fraction of 40%-45% and mild mitral regurgitation. No intracardiac thrombus was documented. In this patient, who had no previous diagnosis of atrial fibrillation and was not receiving anticoagulant therapy, a cardiac source of embolism was considered the most likely etiology. The patient was evaluated by general surgery, urology, internal medicine, and cardiology, and no emergency surgical intervention was recommended. Following radiological confirmation of renal and splenic infarctions and multidisciplinary assessment, anticoagulation with subcutaneous enoxaparin at a dose of 1 mg/kg every 12 hours was initiated. 

The patient was referred to a tertiary care center for further etiological evaluation and long-term anticoagulation planning. Follow-up through the national electronic health record system after referral revealed that peripheral blood smear, coagulation tests, hemoglobin electrophoresis, and investigations for sickle cell disease performed at the tertiary care hospital showed no pathological findings. The patient’s symptoms completely resolved on the ninth day of treatment, and she was discharged in good clinical condition after completing a 14-day course of therapy. Long-term anticoagulation with subcutaneous enoxaparin at 1 mg/kg every 24 hours was continued after discharge.

## Discussion

This case demonstrates a rare but clinically important presentation of newly diagnosed atrial fibrillation: simultaneous renal and splenic infarctions. Although atrial fibrillation is most commonly recognized as a risk factor for cerebral embolic events, it may also cause systemic embolization involving visceral organs [8]. Concurrent renal and splenic infarctions suggest a systemic embolic process rather than an isolated local vascular event [2].

Previous reports of simultaneous renal and splenic infarctions have described various underlying mechanisms, including hypercoagulable states, infection-related thrombosis, aortic pathology, and cardioembolic disease [1,2,9]. In contrast, the present case is notable because the visceral infarctions were detected during the initial emergency department evaluation of non-specific abdominal, back, and flank pain, and newly diagnosed atrial fibrillation was identified in the absence of significant renal or splenic arterial stenosis, aortic mural thrombus, or relevant aortic plaque formation on CT angiography. These findings support a probable cardioembolic mechanism and emphasize the need to consider alternative embolic and hypercoagulable causes in similar presentations.

The clinical diagnosis of renal and splenic infarctions is challenging because symptoms are often nonspecific. Renal infarction may present with flank, abdominal, or back pain and can mimic renal colic or acute pyelonephritis [2]. Similarly, splenic infarction may present with left upper quadrant pain, diffuse abdominal pain, nausea, vomiting, fever, or non-specific systemic symptoms [3]. In the present case, the patient presented with epigastric, back, and flank pain, and the initial clinical picture could have been confused with more common abdominal or urinary conditions.

Laboratory findings may provide important diagnostic clues. Elevated LDH is frequently reported in renal infarction and may reflect tissue ischemia, although it is not specific [4]. In our patient, LDH was markedly elevated despite preserved renal function. Mild leukocytosis, neutrophilia, elevated CRP, and slightly elevated D-dimer were also observed. Urinalysis did not show strong evidence of urinary tract infection, and no urinary stone or hydronephrosis was detected on imaging. These findings supported the need for contrast-enhanced imaging.

Contrast-enhanced CT is the key diagnostic modality for suspected visceral infarction [3,6]. In this patient, contrast-enhanced CT demonstrated non-enhancing lesions in both the spleen and right kidney. Subsequent CT angiography did not show significant stenosis of the splenic or renal arteries, mural thrombus, or relevant aortic plaque formation. The absence of major arterial obstruction or significant local vascular pathology supported a probable cardioembolic mechanism, particularly in the setting of newly diagnosed atrial fibrillation.

The pathophysiological link between atrial fibrillation and systemic embolization is well established. Atrial fibrillation promotes thrombus formation through atrial blood stasis, endothelial dysfunction, and activation of coagulation pathways [10]. Although stroke is the most feared embolic complication, emboli may also lodge in visceral arterial branches and cause renal, splenic, or mesenteric infarction [8]. In patients with simultaneous visceral infarctions, other causes such as infective endocarditis, antiphospholipid syndrome, vasculitis, malignancy, myeloproliferative disorders, aortic mural thrombus, and infection-related hypercoagulability should also be considered [11]. In the present case, newly detected atrial fibrillation and the absence of significant arterial stenosis or mural thrombus on CT angiography made cardioembolism the most plausible mechanism.

The optimal treatment of acute renal and splenic infarctions is not fully standardized and should be individualized according to the timing of diagnosis, extent of infarction, renal function, hemodynamic status, suspected embolic source, and bleeding risk [2,11]. In clinically stable patients with a cardioembolic source, systemic anticoagulation is generally the main therapeutic approach [6,11]. Endovascular revascularization, catheter-directed thrombolysis, or thrombectomy may be considered in selected patients, particularly when diagnosis is made early or when extensive renal ischemia, main renal artery involvement, solitary kidney, bilateral disease, or progressive renal dysfunction is present [6,11]. Surgical intervention is usually reserved for complications such as splenic rupture, abscess, hemorrhage, or clinical deterioration [2,11]. In this case, no emergency surgical intervention was indicated, and anticoagulation with enoxaparin was initiated. Because the patient had a recent history of spontaneous ecchymoses, further multidisciplinary assessment was recommended to balance thromboembolic and bleeding risks during long-term anticoagulation planning.

## Conclusions

Simultaneous renal and splenic infarctions are rare but clinically important manifestations of systemic embolization. Newly diagnosed atrial fibrillation may be associated with visceral embolic events and should be considered as a possible cardioembolic source, particularly when no significant local arterial obstruction or aortic thrombus is identified. In patients presenting with unexplained acute abdominal, back, or flank pain, the coexistence of elevated LDH and atrial fibrillation should prompt consideration of renal and splenic infarctions. Contrast-enhanced CT plays a central role in timely diagnosis. Anticoagulation should be considered on an individualized basis, taking into account the suspected etiology, infarct extent, bleeding risk, and multidisciplinary clinical assessment.
